# Assessment of Caries Risk Through Clinical and Salivary Parameters in Pediatric Patients with Cleft Lip and Palate

**DOI:** 10.3390/pediatric18040113

**Published:** 2026-08-14

**Authors:** Alícia Lima, Anabela Paula, Catarina Nunes, Raquel Travassos, Bárbara Oliveiros, Carlos Miguel Marto, Eunice Carrilho, Inês Francisco, Francisco Vale

**Affiliations:** 1 Institute of Integrated Clinical Practice, Faculty of Medicine, University of Coimbra, 3000-370 Coimbra, Portugal; lima_alicia@hotmail.com (A.L.); cmiguel.marto@uc.pt (C.M.M.); eunicecarrilho@gmail.com (E.C.); 2 Institute of Orthodontics, Faculty of Medicine, University of Coimbra, 3000-370 Coimbra, Portugal; mcal9497@hotmail.com (C.N.); raqueltravassos.91@gmail.com (R.T.); ines70.francisco@gmail.com (I.F.); fvale@fmed.uc.pt (F.V.); 3 Laboratory of Evidence-Based and Precision Dentistry, Faculty of Medicine, University of Coimbra, 3000-075 Coimbra, Portugal; boliveiros@fmed.uc.pt; 4 Coimbra Institute for Clinical and Biomedical Research (iCBR), Area of Environment, Genetics and Oncobiology (CIMAGO), Faculty of Medicine, University of Coimbra, 3000-548 Coimbra, Portugal; 5 Centre for Innovative Biomedicine and Biotechnology (CIBB), University of Coimbra, 3004-504 Coimbra, Portugal; 6 Clinical Academic Center of Coimbra (CACC), 3004–504 Coimbra, Portugal; 7 Centre for Mechanical Engineering, Materials and Processes (CEMMPRE), Advanced Production and Intelligent Systems (ARISE), Department of Mechanical Engineering, University of Coimbra, 3030-788 Coimbra, Portugal; 8 Laboratory of Biostatistics and Medical Informatics, Faculty of Medicine, University of Coimbra, 3000-548 Coimbra, Portugal; 9 Institute of Experimental Pathology, Faculty of Medicine, University of Coimbra, 3000-548 Coimbra, Portugal

**Keywords:** dental caries, cleft palate, cleft lip, cleft lip and palate, DMFT index, orthodontics

## Abstract

Objective: The aim of this study is to assess caries-related indicators (DMFT and ICDAS indices), plaque index, salivary pH and buffering capacity, and dietary and oral hygiene habits in patients with cleft lip and/or palate undergoing orthodontic treatment, compared with a control group of healthy patients. Methods: This pilot case–control study included 26 patients undergoing orthodontic treatment, 13 patients with cleft lip and/or palate and 13 healthy individuals. The following variables were assessed: diet and oral hygiene through a questionnaire; salivary pH and buffering capacity; caries risk, using the DMFT and ICDAS indices; and plaque index, using the ImageJ Software. Results: No statistically significant differences were detected in the DMFT index, salivary pH, and plaque index; nor in the diet, oral hygiene and salivary buffering capacity. A slightly higher plaque index was observed in patients with cleft lip and/or palate compared to healthy patients. There was a statistically significant difference in the location of plaque, with a higher incidence in the upper central incisors in the study group. Conclusion: This pilot study suggests no statistically significant differences in the DMFT index, plaque index, salivary pH, and buffering capacity between patients with cleft lip and/or palate and healthy controls undergoing orthodontic treatment. However, a higher incidence of bacterial plaque on the upper central incisors was observed in patients with cleft lip and/or palate.

## 1. Introduction

Dental caries pathology is a dynamic process of demineralization and remineralization that occurs on the tooth surface, involving several factors, including microbial, genetic, immunological, behavioural, and environmental factors [1]. Dental caries results from the action of various bacteria that can lead to partial or total tooth destruction. *Streptococcus* species, namely *Streptococcus mutans* and *Streptococcus sobrinus*, are the main etiological agents of dental caries. Gram-positive bacilli, such as *Lactobacillus*, *Eubacterium*, and *Propionibacterium*, are late colonizers of dental caries, in the deep area of the lesion [2].

The oral cavity provides a favourable environment for the development of diverse oral microbial communities. The complex anatomy of the oral cavity and its interaction with the external environment contribute to the complexity and ecological specificity of the microbiome present. The diversity and development of the oral microbiota are influenced by endogenous and exogenous factors that maintain it in dynamic balance. Dysbiosis of this oral microbiota, characterized by a disruption in the microecological balance between the host and microorganisms, can trigger oral infectious diseases such as dental caries, among others, thus determining oral health status [3].

The literature has suggested that patients with cleft lip and/or palate (CLP) have a different oral microbiota compared to individuals with normal orofacial development, determining, among other factors, a higher prevalence of caries lesions [4,5,6,7,8,9]. Bokhout et al. recorded up to 3.5 times more carious tooth surfaces in children with CLP than in the control group without CLP, with this difference being most obvious in the primary dentition [10].

CLP is a congenital craniofacial malformation that significantly impacts the structure and function of the oral cavity, resulting in alterations in facial features, such as an abnormal bone and nasal structure, often accompanied by malformations of the oral muscles [3,6,11,12,13,14,15,16]. These anomalies occur during fetal development and result from the failure of fusion of the lateral and median nasal processes, part of the frontonasal prominence, and the maxillary prominence [7,17]. It has a worldwide incidence of approximately 0.15% per birth. However, it is recognized that this number may vary among different populations and may be associated with ethnic origin, genetic factors, environmental conditions, and gender [7,13].

Patients with CLP may also face significant functional challenges, including problems with speech, breathing, sucking, chewing, swallowing, and social integration. Therefore, they require long-term multidisciplinary rehabilitation, which should begin at birth and continue into adulthood [3,5,6,13].

Orofacial clefts are classified into three main categories: cleft lip (CL); cleft palate (CP); and cleft lip and/or palate. Cleft classification is based on embryonic development, considering the cause and extent of physical involvement. Regarding etiology, clefts can be distinguished between syndromic and non-syndromic. The term “non-syndromic” refers to the absence of physical or developmental abnormalities and the lack of known teratogenic exposure causing CLP. Based on the extent of tissue involvement, CLP can be classified as unilateral or bilateral and incomplete or complete. Unilateral clefts affect only one side of the face, while bilateral clefts involve both sides. Incomplete CLP involve the lip and anterior maxilla, while complete CLP include the lip, anterior maxilla, and hard and soft palate [17,18,19].

According to the literature, several factors can increase the risk of caries in patients with CLP. These patients are often born into more disadvantaged socioeconomic backgrounds compared to patients without CLP; and many parents or caregivers of children with CLP have low oral health literacy and limited contact with healthcare professionals. Furthermore, they have inadequate oral hygiene practices, with difficulty cleaning the teeth adjacent to the cleft at an early age due to the loss of elasticity of the surgically repaired lip, the anatomy of the cleft, and a fear of brushing the cleft area. Other factors may contribute to the risk of caries, namely increased dietary carbohydrate consumption and feeding difficulties in children with CLP, associated with prolonged periods of bottle-feeding, which often contain a higher sugar content compared to breastfeeding [4,5,6,7,20,21,22,23,24]. Additionally, oronasal communication may promote greater adhesion of bacterial plaque to the teeth due to the viscosity of nasal fluid [4,6]. The presence of CLP is also associated with areas of stagnation and promotion of dysbiotic plaque biofilm [4,7].

Patients with CLP also frequently present with dental anomalies, such as enamel hypoplasia, hypodontia, and abnormal tooth size and shape; and skeletal anomalies, such as supernumerary teeth, discrepancies in skeletal base relationships, and dental crowding, promoting increased plaque retention and greater difficulty in cleaning [4,25,26]. In most cases, these patients are indicated for orthodontic treatment integrated with multidisciplinary rehabilitation. However, the presence of an orthodontic appliance can favor the colonization of cariogenic bacteria, such as *Streptococcus mutans*, and late colonizers, such as *Lactobacillus*, increasing the predisposition to dental caries. The combination of these factors will, as mentioned, cause increased bacterial colonization, which predisposes to the development of dental caries, complicating and prolonging surgical and orthodontic treatment [3,4,27,28]. Other factors, such as reduced salivary flow and the increased prevalence of mouth breathing in patients with CLP, are also considered risk factors for the development of dental caries [7,29,30,31].

Although it has been previously established that the oral microbiome of patients with CLP differs from that of non-carriers, the precise nature of this variation, including the relevant bacterial species, has not been fully elucidated [6,7]. Studies indicate the presence of significantly higher levels of *Streptococcus mutans*, *Lactobacillus*, *Prevotella*, and *Candida albicans* in patients with CLP compared to the control group [10]. However, the available literature can present controversial results, which may be due to the multifactorial aetiology of dental caries, cultural variations, differences in health literacy, anatomical variation in patients’ oral cavity, different types and anatomical locations of the cleft, differences in the various methodologies used in the research, and heterogeneity of the study groups. There are references in the literature that the type and location of the cleft can, respectively, influence the extent and location of caries [3,4,7].

Given the multiple factors that have been reported to affect caries risk in patients with CLP, it has been hypothesized in the literature that these patients present greater susceptibility to caries compared to individuals without this condition, although results across studies remain inconsistent [4,7]. Given these considerations, it is crucial to prioritize the provision of consistent and comprehensive oral health care to patients with CLP, aiming to mitigate the increased caries risk and incidence observed in these patients. Therefore, it is important to educate parents and healthcare professionals about the increased predisposition to caries in patients with CLP. This involves educating patients and caregivers about the importance of good oral hygiene practices, promoting awareness of dietary habits that promote oral health, and implementing individualized preventive strategies. It is essential to prioritize primary preventive oral health approaches in patients with CLP from an early age, especially those at high risk for dental caries and undergoing orthodontic treatment [3,4].

Despite previously published results, there are gaps in the literature, and a better understanding of the differences in the oral flora of patients with CLP undergoing orthodontic treatment and their risk of caries is needed. This research aims to assess the risk of caries in patients with CLP undergoing orthodontic treatment. This research aims to evaluate caries-related indicators (DMFT and ICDAS indices) and plaque index, salivary pH and buffering capacity, and dietary and oral hygiene habits in patients with CLP undergoing orthodontic treatment, compared to a control group without CLP [7].

## 2. Materials and Methods

### 2.1. Participant Selection

Patient recruitment for this case–control clinical study was conducted by 2 researchers, selecting patients from the Orthodontics clinic of the Orthodontics Institute of the Faculty of Medicine of the University of Coimbra (FMUC). After selection, each individual was identified by a code specifically created for this study. All participants were treated with fixed orthodontic appliances of the same type and commercial brand (Roth prescription, 0.018-inch slot brackets), with standardized archwires and ligatures.

The sample selection considered the following inclusion and exclusion criteria:

- Inclusion criteria—patients from the Orthodontics clinic of the Orthodontics Institute of the FMUC, of both sexes, wearing fixed orthodontic appliances, aged between 5 and 15 years; for the control group, systemically healthy patients without cleft lip and/or palate; for the study group, patients with surgically corrected cleft lip and/or palate.
- Exclusion criteria—syndromic patients or patients with craniofacial anomalies other than CLP; patients with a history of craniofacial trauma, metabolic, or endocrine diseases.

After applying the inclusion and exclusion criteria, the sample was divided into two groups:

- Control group—composed of healthy patients undergoing treatment with fixed orthodontic appliances.
- Study group—composed of patients with surgically corrected cleft lip and/or palate undergoing treatment with fixed orthodontic appliances.

This study was conducted in accordance with the Declaration of Helsinki and received a favourable opinion from the Ethics Committee of the Faculty of Medicine of the University of Coimbra on 8 January 2024, with process identification number CE-175/2023. No a priori sample size calculation was performed; the final sample reflects the number of eligible patients meeting the inclusion criteria who were available for recruitment during the study period, given the low incidence of CLP and the additional requirement of active fixed orthodontic treatment at a single clinical centre.

### 2.2. Study Design

After sample selection, the following variables were assessed: dietary and oral hygiene habits; salivary pH and buffering capacity; caries risk using the DMFT and ICDAS (International Caries Detection and Assessment System) indices; and plaque index.

### 2.3. Data Collection

First, patients and their legal guardians were asked to complete a questionnaire on dietary and oral hygiene habits. All clinical examinations and salivary assessments were performed between 6 and 12 months after the initiation of orthodontic treatment, corresponding to the alignment and levelling phase. This standardized timing was adopted to minimize the potential in-fluence of treatment duration on plaque accumulation and caries-related variables.

**Figure 1:**
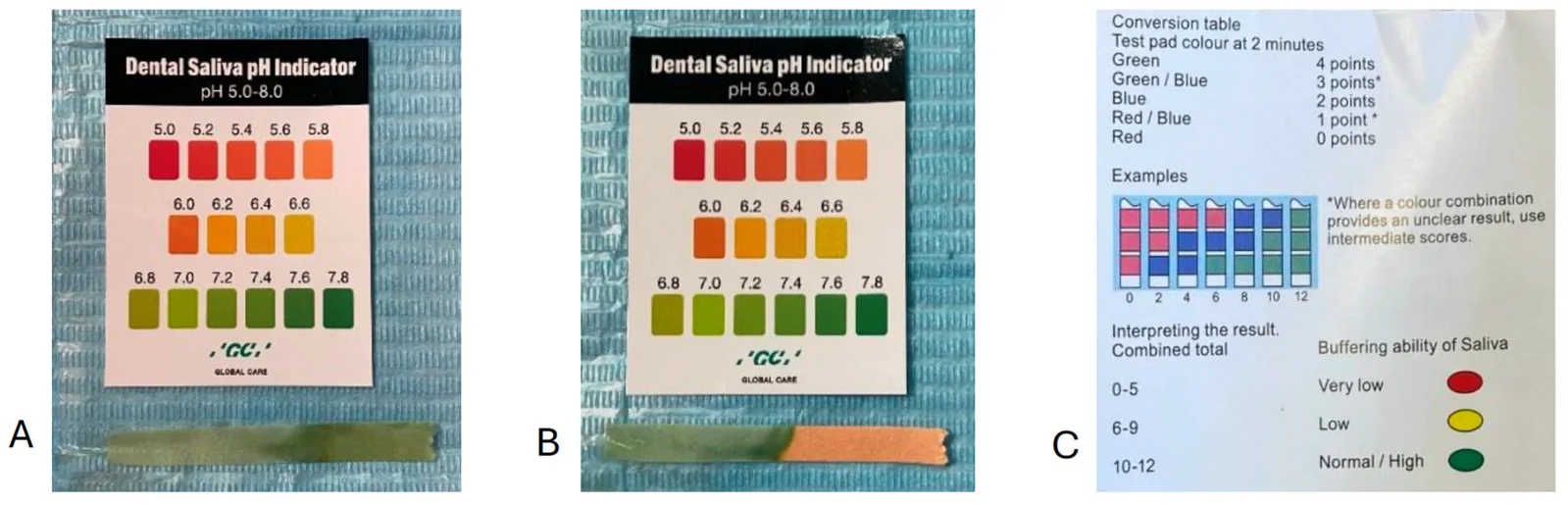
Image illustrating pH measurement with pH strips (GC Dental Saliva Ph Indicator^®^) in a patient from the study group (**A**) and in a patient from the control group (**B**); and instructions for calculating buffer capacity using GC Saliva-Check Buffer kit^®^ (**C**).

Subsequently, 3 mL of unstimulated saliva was collected by asking the patient to expel saliva into a sterile 5 mL Eppendorf tube. Two measurements were then performed: salivary pH and buffering capacity. Patients were instructed to abstain from eating for at least 2 h prior to saliva collection and to avoid consuming any beverages other than water during this period. They were also instructed to abstain from any oral hygiene procedures (toothbrushing, flossing, or mouthwash use) for at least 2 h before sampling. Although collection time depended on individual appointment scheduling, all saliva samples were collected during morning appointments between 9:00 and 11:00 a.m. Unstimulated saliva samples (3 mL) were collected within a maximum of 5 min per patient. Salivary pH was measured using GC Dental Saliva pH Indicator^®^ pH test strips. (Figure 1A,B). Measurements that did not produce a clear and interpretable colorimetric reaction were considered invalid and excluded from the analysis.

Buffer capacity was measured with the GC Saliva-Check Buffer kit^®^ (GC Corporation, Tokyo, Japan), following the manufacturer’s instructions (Figure 1C).

The oral clinical examination of the patients was performed by a calibrated researcher in a dental office under ideal conditions. The caries indexes were completed: DMFT and ICDAS. GC Tri Plaque ID Gel^®^ plaque developer was applied, and intraoral frontal photographs were taken. The plaque index was calculated using ImageJ (version 1.54; National Institutes of Health, Bethesda, MD, USA) from these photographs. Photographs were standardized frontal images of the four maxillary anterior teeth (central and lateral incisors, when present), captured with the same equipment (Canon EOS R50; Canon RF 85 mm F2 Macro IS STM lens; Macro Ring Flash MF-R76) under fixed manual settings (F29, flash 1:4, ISO 200, shutter speed 1/160 s) and consistent ambient lighting. The region of interest was manually delimited using ImageJ’s Freehand Selection tool, followed by color threshold conversion (B&W, HSB, dark background) and saturation adjustment to isolate plaque-disclosed areas; the plaque index was calculated as the percentage of stained (white) pixels relative to total pixels within the region of interest.

**Figure 2:**
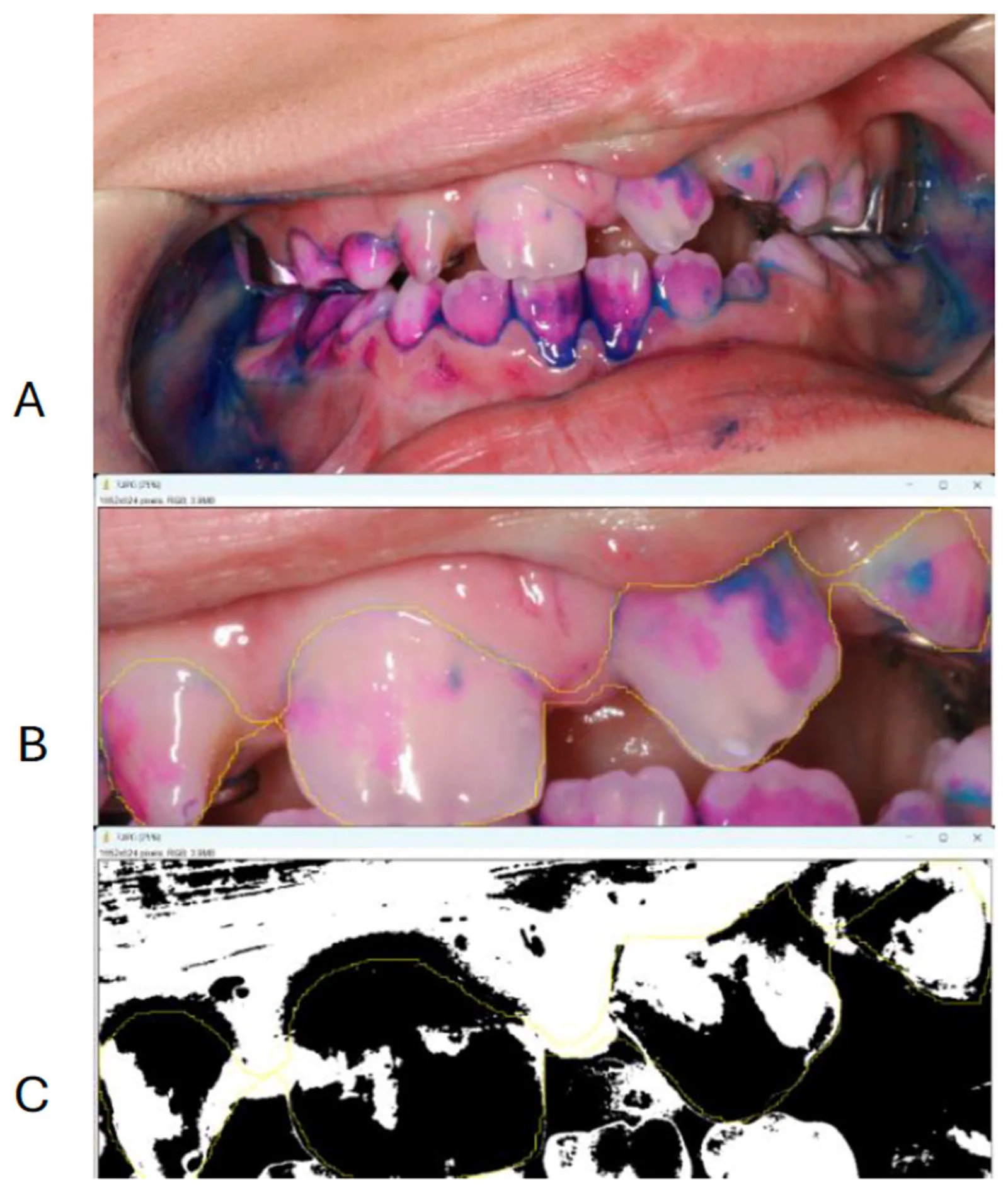
Analysis of the plaque index using the ImageJ program. (**A**) delimitation of the region of interest; (**B**) cropping of the image of the region of interest; (**C**) after adjusting the colour to black and white and varying the saturation, leaving the dental surfaces with plaque developer in white.

Image processing was performed as shown in Figure 2. Each original image was imported and processed in ImageJ.

ICDAS was recorded during the clinical examination to characterize lesion severity at the tooth-surface level, complementing the DMFT index; however, given the small sample size, ICDAS scores were not subjected to separate inferential statistical comparison between groups, as the resulting subgroup frequencies per code would be too sparse for reliable analysis. ICDAS data are therefore reported descriptively only where relevant, and DMFT was used as the primary outcome for between-group statistical comparison.

### 2.4. Statistical Analysis

Statistical analysis of the data was performed using the IBM^®^ SPSS^®^ Statistics platform (version 29), with a statistical significance level of 0.05. Two types of variables were analysed: quantitative and qualitative.

The quantitative variables analysed were: age, salivary pH, plaque index, decayed teeth, missing teeth due to decay, filled teeth, and the total DMFT index score. Statistical analyses of salivary pH were performed using the available valid observations only (available-case analysis). Three statistical tests were used according to distribution and homogeneity: Student’s *t*-test, Welch’s *t*-test, and Mann–Whitney U test. Normality was assessed using the Shapiro–Wilk test. If the results were negative (Shapiro–Wilk *p* < 0.05), with a non-normal distribution, the *p*-value of the Mann–Whitney U test was applied. If the distribution was normal (Shapiro–Wilk *p* > 0.05), the homogeneity of variances between groups was assessed using Levene’s test. When homogeneity was found (Levene’s test *p* > 0.05), Student’s *t*-test *p*-value was applied; if not (*p* < 0.05 in Levene’s test), the Welch *t*-test *p*-value was applied.

The qualitative variables analysed were: sex; systemic pathologies; drug therapy; allergies; cleft classification; family history; eating habits such as meal times (breakfast, mid-morning snack, lunch, afternoon snack, dinner, and supper) and the frequency of eating certain foods (milk, yogurt, cheese, butter; bread; potatoes; green vegetables; fruit; fruit juices; rice; legumes; meat; eggs; fish; soup; cakes; sweets; soft drinks; coffee/tea with sugar; and chewing gum); Oral hygiene habits (brushing frequency, toothbrush type, use of dental floss, toothbrushes, or mouthwash, parental assistance/supervision, and use of fluoride supplements); salivary buffering capacity; location of plaque collection; and dentition type. For qualitative variables, the association of these variables with the study groups was assessed using Fisher’s exact test. When an association occurred (Fisher’s exact test *p* < 0.05), we sought to evaluate residuals greater than 1.96 in absolute value to assess trends. Given the exploratory nature of this pilot study and the large number of qualitative variables compared, no correction for multiple comparisons (e.g., Bonferroni) was applied, as doing so would have further reduced statistical power in an already small sample. Consequently, all statistical comparisons in this study should be interpreted as exploratory and hypothesis-generating rather than confirmatory.

## 3. Results

### 3.1. Sample Characterization

The sample includes healthy patients of both sexes, with surgically corrected cleft lip and palate undergoing orthodontic treatment with fixed appliances, aged between 5 and 15 years (Table 1).

Analysis of quantitative variables Table 2 presents the mean, standard deviation, minimum and maximum values, percentiles, and *p*-value for the variables: age, salivary pH, plaque index, decayed teeth, teeth lost due to caries, filled teeth, and total DMFT index score. Salivary pH analysis was based on the available valid observations, as seven measurements in the control group were excluded because they did not produce a clear and interpretable colorimetric reaction. Analysis of Table 2 shows that there are no statistically significant differences (*p* > 0.05) in the aforementioned variables between the study group and the control group.

Table 3 and Table 4 presents, in greater detail, the calculation of the plaque index using ImageJ software, in the control group and in the study group, respectively. In the control group, an average of 23.4% was obtained, with a maximum value of 42.2% and a minimum value of 10.1%. In the study group, there was an average of 31.8%, with a maximum value of 53.4% and a minimum value of 1.2%. No statistically significant difference was found in the plaque index in both groups.

### 3.2. Analysis of Qualitative Variables

The statistical analysis of the qualitative variables evaluated: sex; systemic pathologies; drug therapy; allergies; cleft classification; family history; eating habits such as meal times (breakfast, morning snack, lunch, snack, dinner and supper) and the frequency of intake of certain foods (milk, yogurt, cheese, butter; bread; potatoes; green vegetables; fruits; fruit juices; rice; legumes; meat; eggs; fish; soup; cakes; sweets; soft drinks; coffee/tea with sugar and chewing gum); oral hygiene habits (frequency of brushing, type of brush, use of dental floss, toothbrushes or mouthwash, parental help/supervision and use of fluoride supplement); salivary buffering capacity; location of bacterial plaque collection; and type of dentition. There are only statistically significant differences in the classification of the cleft (Fisher, *p* < 0.001), with a higher prevalence of patients with unilateral CLP in the study group; in family history (Fisher, *p* = 0.039), with a predominance in the study group; in the frequency of potato intake (Fisher, *p* = 0.034), with consumption being more common in the study group (2–3×/week); and in the location of bacterial plaque collection (Fisher, *p* = 0.003), with a higher incidence in the upper central incisors in the study group and on the lingual surface of the lower molars in the control group. No statistically significant differences were observed in the other variables.

## 4. Discussion

This study evaluated the risk of caries in patients with CLP undergoing orthodontic treatment. Secondary objectives included assessing the influence of dietary habits, oral hygiene habits, pH, salivary buffering capacity, and plaque index on caries risk. The use of fluoride supplementation and the location of bacterial plaque were also compared. Several factors can increase the risk of caries in patients with CLP, such as socioeconomic context, inadequate oral hygiene, a diet richer in carbohydrates and sugar, the presence of dental anomalies such as dental crowding and hypodontia, and the use of fixed orthodontic appliances [4,5,6,7,20,21,22,23,24]. It becomes relevant to evaluate these factors in order to improve the understanding of the association between CLP and caries risk.

In the present study, no statistically significant differences (*p* = 0.248) were recorded in the risk of dental caries between the study group (0.209 ± 0.2489) and the control group (0.124 ± 0.1252), as assessed by the DMFT index. There were also no statistically significant differences in the values of the DMFT index constituents (decayed teeth, teeth missing due to caries, and filled teeth). Consistent with these results, Lucas et al. reported no statistically significant differences in the DMFT indices between the two groups—1.18 ± 1.67 and 2.35 ± 3.38 in patients with CLP, respectively, and 1.48 ± 2.33 and 2.93 ± 3.14 in control patients—and also found a greater number of filled dental surfaces in patients in the control group, possibly because the families of patients with CLP are more concerned with other aspects of general health, not prioritizing oral health [32]. Sundell et al., in a study comparing the salivary microbiological profile of 5-year-old children, found no statistically significant differences in the DMFT index of patients with CLP—1.2 ± 2.5 compared to 0.9 ± 3.0 in the control group [33]. However, a systematic review by Grewcock et al. reported a greater experience of caries in the deciduous and mixed dentition in patients with CLP compared to healthy patients [7]. Similarly, Kirchberg et al. stated that patients with CLP have fewer healthy teeth, with the prevalence of caries in the deciduous dentition of patients aged 6–7 years being twice as high compared to healthy patients; and several teeth lost due to caries being twice as high in patients with CLP [34]. A similar result was also observed in a Syrian population by Al-Dajani et al., where 85% of patients with CLP had a moderate or high risk of caries compared to 45.3% of their healthy siblings [35]. These contradictory results may be attributed to several factors such as the size and selection of the study samples, the race studied, the geographical distribution, age, and the use of different methodologies [36,37]. An important aspect that must be considered when interpreting the absence of statistically significant differences in caries risk between groups is that all participants, in both the study and control groups, were undergoing treatment with fixed orthodontic appliances. Fixed appliances are independently associated with increased plaque retention and cariogenic bacterial colonization, regardless of underlying craniofacial anatomy. It is therefore plausible that this shared risk factor exerted a comparable effect on both groups, effectively leveling baseline plaque accumulation and caries risk, and potentially masking a CLP-specific effect that might be more apparent in a non-orthodontic population. This interpretation is further supported by the fact that, although overall caries risk and plaque index did not differ significantly between groups, the location of plaque accumulation did differ significantly (Fisher, *p* = 0.003), with a higher incidence in the upper central incisors—adjacent to the cleft region—in the study group. This suggests that the CLP-specific effect on oral hygiene may manifest primarily as a localized, anatomy-driven pattern of plaque accumulation rather than as an overall increase in caries burden, particularly when both groups are already subject to the elevated baseline risk associated with fixed orthodontic treatment. Future studies comparing CLP and non-CLP patients without concurrent orthodontic treatment would be necessary to isolate the independent effect of the cleft condition on caries risk.

Regarding dietary habits, there were no statistically significant differences in either the timing and number of meals or the frequency of food intake evaluated. The only noteworthy difference is the statistically significant difference (Fisher, *p* = 0.034) in the frequency (2–3×/week) of potato consumption by the study group. Given the relatively small sample size and the large number of statistical comparisons performed, this isolated finding should be interpreted with caution and considered exploratory rather than indicative of a clinically meaningful association. These results are consistent with the study by Sundell et al. and the study by Bokhout et al., which found no statistically significant differences in the diet and frequency of meals in patients with CLP compared to healthy patients [33,38]. However, Parapanisiou et al. reported that 7.3% of patients with CLP and 4.9% of healthy patients consume juices and soft drinks more than 3 times a day, while 61% of patients in both groups consume them 1 to 3 times a day. They also found that patients in both groups have an average of 3 main meals and 2 secondary meals per day, data supported by this study [36]. Allam et al. analysed the deciduous and mixed dentition of patients with CLP and observed a direct correlation between caries and consumption of foods/drinks rich in sugar and carbohydrates between meals, but no correlation was found between the risk of caries and the consumption of these foods during meals. This difference may be related to the increased salivary flow caused by chewing; with the presence of protective components in the diet, such as calcium, phosphates, fats, proteins and fluorides; or with reinforced oral hygiene habits that ultimately balance this correlation [39].

Regarding oral hygiene habits, no statistically significant differences were recorded in brushing frequency; type of brush; use of dental floss, interdental brushes or mouthwash; parental help/supervision and use of fluoride supplements. Parapanisiou et al., using a questionnaire, obtained similar brushing frequencies in patients with and without CLP, with approximately half of the sample brushing twice a day, results like those of this study. They also found that, in the group of patients with CLP, 53.7% of parents supervise their children’s brushing, while in the control group only 39% of parents report supervision. This study also reports that 29% of patients with CLP take fluoride supplements compared to 5% in the control group [36]. Sundell et al. and Bokhout et al., however, found no statistically significant differences in fluoride supplement intake, corroborating the results presented in this study [33,38]. This absence of differences in fluoride supplementation may be due to the increased attention and concern of parents of patients with CLP regarding the prevention of dental caries. On the other hand, there are studies that have found a statistically significant difference in oral hygiene habits in patients with CLP, considering them to have worse oral hygiene compared to healthy patients [4,39,40]. This disparity between studies may be due to cultural differences in the populations studied. To overcome this factor, a study with a multicenter methodology should be carried out.

The results showed no statistically significant difference (*p* = 0.097) in the plaque index between the two groups—0.318 ± 0.1452 in the study group and 0.234 ± 0.0986 in the control group—however, there is a slightly higher index in the study group. Sundell et al. evaluated the plaque index using the Quigley-Hein index—2.2 ± 0.6 in patients with CLP and 2.0 ± 0.7 in the control group—finding no statistically significant differences [33]. In contrast, Veiga et al. reported a higher plaque index in patients with CLP [37]. Similarly, Parapanisiou et al., who calculated the plaque index according to the Silness and Löe criteria, presented similar results [36]. These differences may be due to the different methods used to evaluate the plaque index. The analysis using ImageJ, which was the method used in our study to determine the plaque index, transforms qualitative data into quantitative data in an accurate, specific, and reproducible way. However, given the pilot nature of this study, the observed trend toward higher plaque levels in the CLP group should be interpreted cautiously until confirmed in larger studies.

Regarding the location of bacterial plaque, a statistically significant difference was observed (Fisher, *p* = 0.003) with a higher incidence in the upper central incisors in the study group and on the lingual surface of the lower molars in the control group. This result could be justified by the difficulty that patients with CLP have in cleaning the teeth adjacent to the cleft due to the loss of elasticity of the surgically repaired lip, the anatomy of the cleft, and the fear of brushing the cleft area [4,6,7,20,24]. This finding is biologically plausible and may be explained by the difficulty that patients with CLP experience in cleaning the teeth adjacent to the cleft due to the loss of elasticity of the surgically repaired lip, the anatomy of the cleft, and fear of brushing the cleft area. Nevertheless, given the exploratory nature of this pilot study, this observation should be confirmed in larger studies before definitive conclusions can be drawn.

Regarding the salivary variables evaluated in this study, there were no statistically significant differences in pH (*p* = 0.103) (6.985 ± 0.4120 in the study group and 7.367 ± 0.5279 in the control group) and in buffering capacity (Fisher, *p* = 0.700). These results are in line with the studies by Parapanisiou et al. and Sundell et al., who, in a sample of 133 children with CLP and 297 children without CLP, found no statistically significant difference in salivary buffering capacity between the two groups [36,41]. Talukdar et al. In a sample of 12 patients without CLP and 12 patients with surgically repaired CLP, a significantly lower pH was found in the study group compared to the control group (4.75 ± 0.45 and 7.33 ± 0.49, respectively, with *p* < 0.001) [42]. The divergence in the results of these studies may be due to the different sample sizes. Although the present study yielded findings consistent with those reported in some larger studies, the limited sample size precludes definitive conclusions, and these results should therefore be interpreted with caution until replicated in adequately powered studies.

This study has some limitations such as sample distribution and size, with a reduced number of patients with bilateral CP and CLP and the absence of patients with CL in the study group. This heterogeneity in cleft subtype was not possible to control for or analyze separately given the small sample size, and pooling all CLP subtypes together may obscure clinically relevant anatomical and oral hygiene-related differences between subtypes (e.g., unilateral vs. bilateral, complete vs. incomplete). Future studies with larger, multicentre samples should perform subtype-stratified analyses to clarify whether caries risk and plaque accumulation patterns differ according to cleft type and extent. Thus, it should be considered a pilot case–control study, since the sample size does not allow a representative assessment of the variables evaluated according to the type of cleft. Furthermore, given the large number of statistical comparisons performed relative to the sample size, and the absence of correction for multiple testing, there is an increased risk of false-positive findings. Isolated significant associations, such as the frequency of potato consumption and family history, should therefore be interpreted with particular caution and regarded as exploratory rather than confirmatory; conversely, findings replicated across multiple related variables or supported by biological plausibility (e.g., the localized plaque accumulation adjacent to the cleft region) warrant greater confidence.

Another potential limitation is the variability in the overall duration of orthodontic treatment among participants, particularly because patients with cleft lip and/or palate generally require longer and more complex orthodontic treatment owing to the severity of their malocclusion and the multidisciplinary nature of their care. To minimize this potential confounding factor, all clinical examinations and salivary assessments were performed between 6 and 12 months after the initiation of orthodontic treatment, corresponding to the alignment and leveling phase. Nevertheless, the influence of differences in the overall duration of orthodontic treatment cannot be completely excluded and should be considered when interpreting the present findings.

The use of the ImageJ program also presents some methodological limitations. The gloss on the tooth surface or the image quality of the photos may prevent the program from recognizing surfaces with plaque developer. Additionally, the ImageJ-based plaque index was not validated against a recognized clinical plaque index (e.g., Silness-Löe or Quigley-Hein), image processing was not performed by an examiner blinded to group allocation, and intra- or inter-examiner reliability was not formally assessed. These represent methodological limitations that should be addressed in future studies through examiner blinding, reliability testing, and validation against an established reference index.

Additionally, the wide age range of the sample (5–15 years) encompassed patients in different stages of dentition (primary, mixed, and permanent). The DMFT index, originally designed for permanent dentition, was applied across the whole sample without stratified analysis by dentition stage or combination with the DMFT index for primary teeth. Although dentition type did not differ significantly between groups, minimizing confounding in the between-group comparison, this remains a methodological limitation that should be addressed in future studies with larger, dentition-stratified samples.

Future studies should select a large-scale and homogeneous sample, including patients with various types of cleft lip and palate in the study group. Assessment of salivary flow rate and the study of oral flora microbiology should also be considered to identify the most prevalent group of microorganisms in the oral microbiome of patients with cleft lip and palate, characterizing the microbiological spectrum of the oral cavity. Another aspect of important interest for future studies would be additional evidence on the risk of caries in the permanent dentition in patients with cleft lip and palate, providing a more complete picture of morbidity and oral health-related quality of life due to dental caries in these patients.

## 5. Conclusions

The findings of this pilot study suggest that there are no statistically significant differences in caries risk-related parameters between patients with CLP and patients without CLP undergoing orthodontic treatment. However, a statistically significant difference was observed in the distribution of bacterial plaque, with a higher incidence on the upper central incisors in patients with CLP. This observation is consistent with the recognized difficulty these patients experience in cleaning the teeth adjacent to the cleft region and highlights the potential need for targeted oral hygiene instructions focused on this area.

Future studies should include a larger sample, evaluate the effectiveness of anatomy-specific oral hygiene interventions for the cleft-adjacent region, and conduct a more in-depth investigation of the oral microflora of patients with CLP, encompassing a microbiological characterization of the oral cavity.

## Figures and Tables

**Table 1:** Baseline characteristics of the study participants.

Characteristic	CLP (*n* = 13)	Control (*n* = 13)	*p*-Value
**Age (years), mean ± SD**	10.08 ± 2.99	10.00 ± 2.58	0.979
**Sex, *n* (%)**			
**Male**	8 (61.5%)	6 (46.2%)	
**Female**	5 (38.5%)	7 (53.8%)	

**Table 2:** Descriptive statistics of quantitative variables.

							Percentiles			
	Group	*n*	Mean	Standard Deviation	Minimum	Maximum	25th	50th	75th	*p*
Age	Study	13	10.077	2.9850	7	15	8.0000	8.000	13.000	0.979 ^§^
	Control	13	10.000	2.5820	5	13	8.0000	10.000	12.000	
Salivary pH	Study	13	6.985	0.4120	6.6000	7.800	6.6000	6.800	7.200	0.103
	Control	6	7.367	0.5279	6.6000	7.800	7.0000	7.600	7.750	
Plaque index	Study	13	0.318	0.1452	0.0120	0.534	0.2360	0.288	0.421	0.097 *
	Control	13	0.234	0.0986	0.1010	0.422	0.1850	0.220	0.251	
Decayded teeth	Study	13	3.308	3.4733	0	14	2.0000	2.000	4.000	0.375 ^§^
	Control	13	2.077	1.7059	0	5	1.0000	2.000	3.000	
Teeth lost due to caries	Study	13	0.154	0.3755	0	1	0.0000	0.000	0.000	1.000 ^§^
	Control	13	0.154	0.3755	0	1	0.0000	0.000	0.000	
Filled teeth	Study	13	1.077	1.7541	0	6	0.0000	0.000	1.000	0.376 ^§^
	Control	13	0.615	1.3868	0	5	0.0000	0.000	1.000	
Total DMFT index score	Study	13	0.209	0.2489	0.0380	1.000	0.0870	0.143	0.217	0.248 ^§^
	Control	13	0.124	0.1252	0.0000	0.429	0.0360	0.107	0.143	

* Student’s *t*-test; ^§^ Mann–Whitney U test.

**Table 3:** Calculation of the control group plaque index using ImageJ software.

Sample Code	Original Photo	Cutout	Photo Black/White	Value 0	Value 255	Plaque Index (%)
**11**	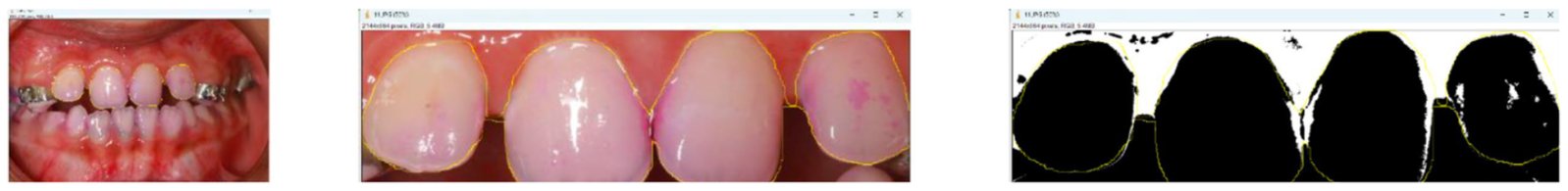			1,159,981	263,635	18.5
**13**	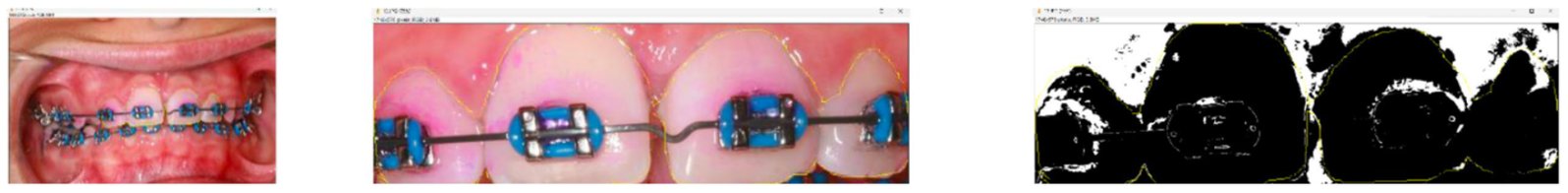			785,461	221,387	22
**15**	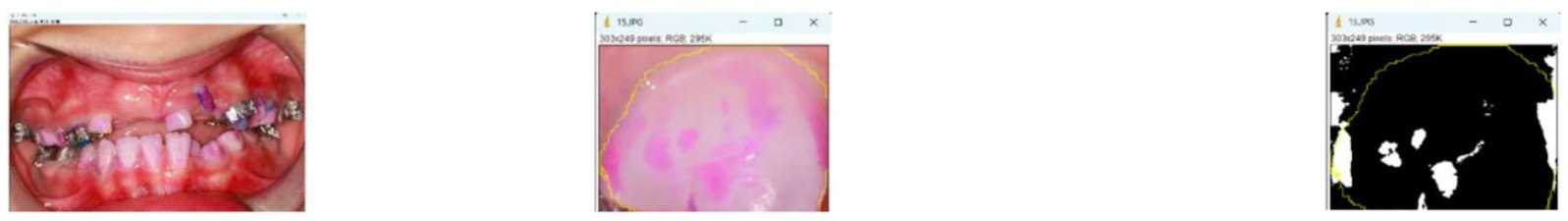			67,511	7936	42
**17**	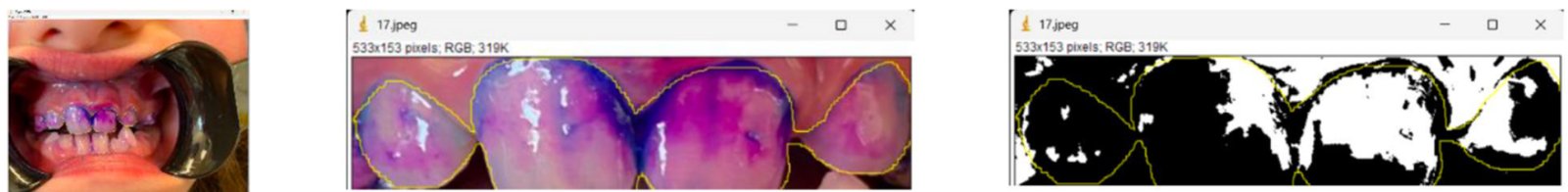			57,805	23,744	29.1
**18**	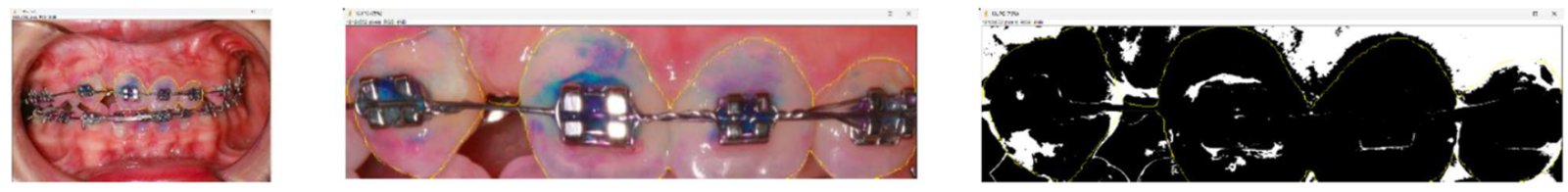			848,765	206,659	19.6
**20**	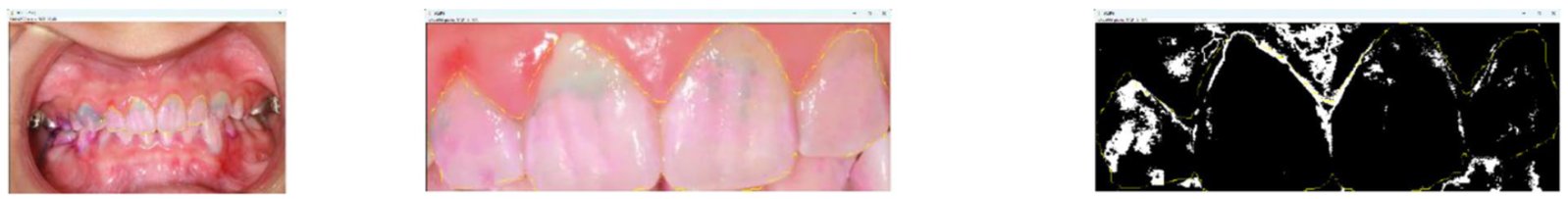			731,708	82,276	10.1
**21**	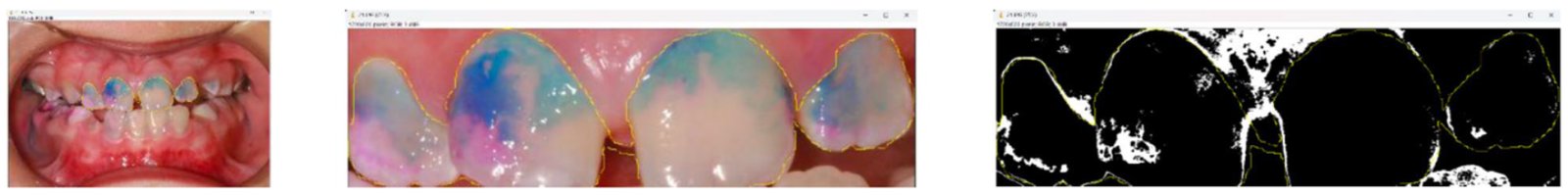			801,209	93,191	10.4
**22**	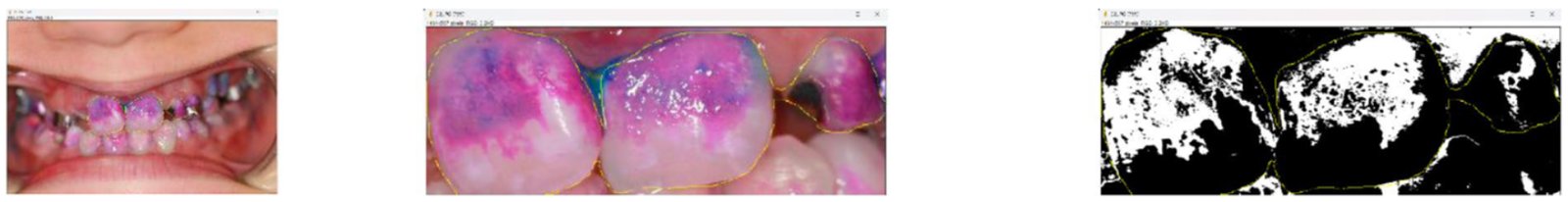			566,174	262,213	42.2
**23**	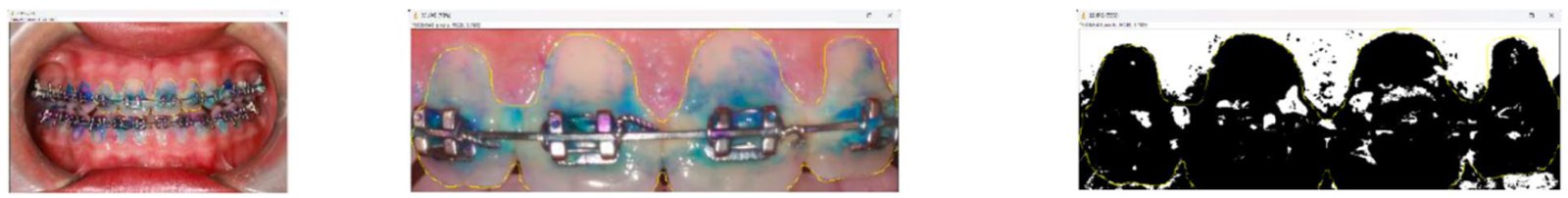			609,925	204,395	25.1
**24**	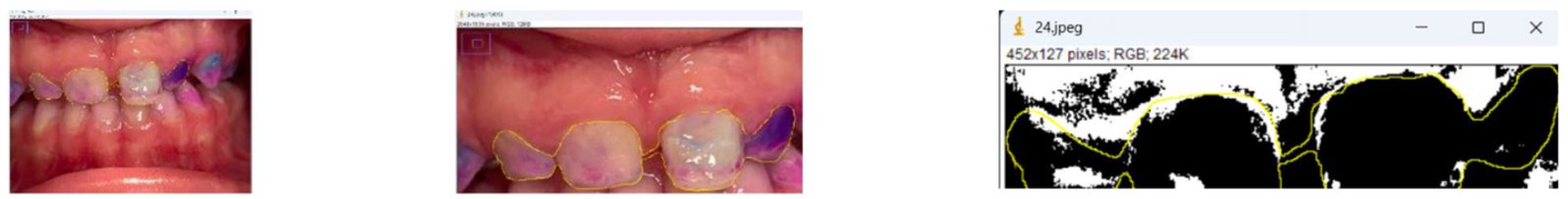			43,704	13,700	23.9
**25**	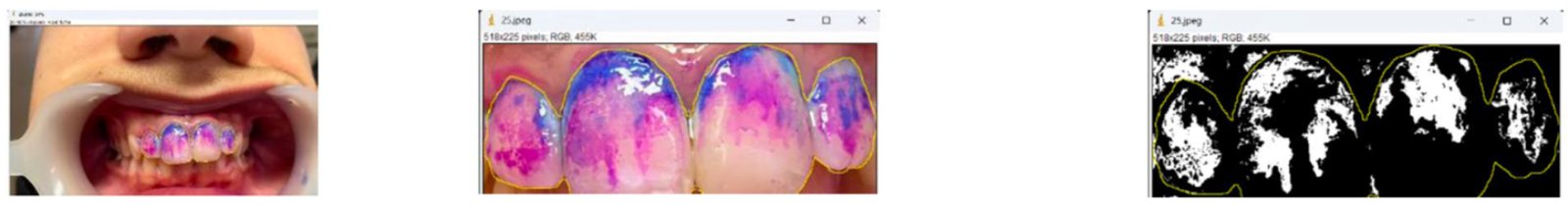			92,681	23,869	20.5
**26**	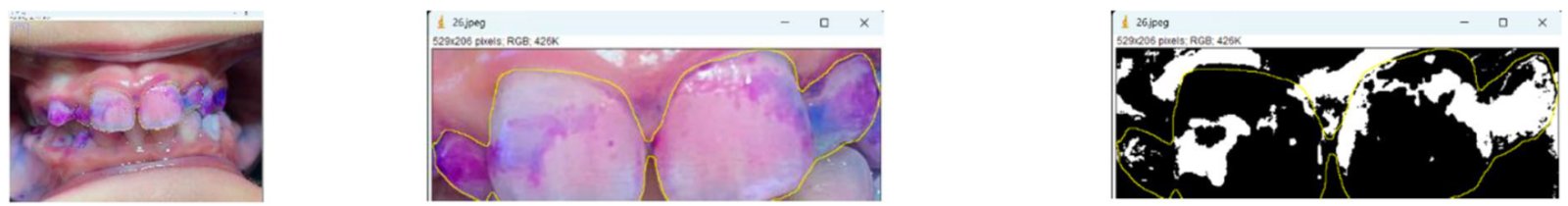			83,764	25,210	23.1
**27**	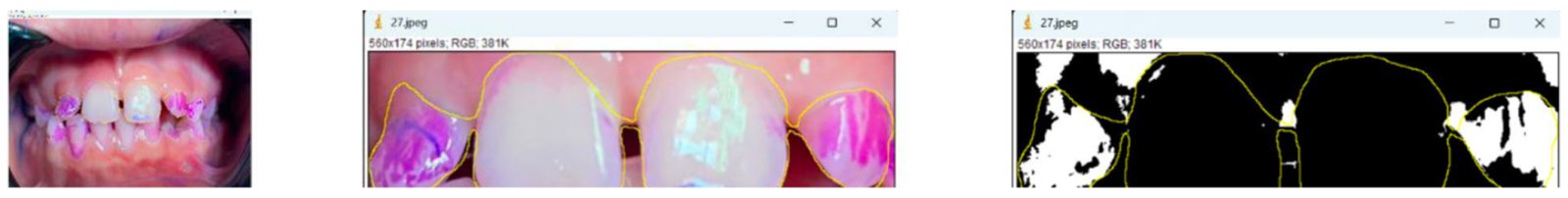			80,442	16,998	17.4

**Table 4:** Calculation of the plaque index of the study group using ImageJ software.

Sample Code	Original Photo	Cutout	Photo Black/White	Value 0	Value 255	Plaque Index (%)
**1**	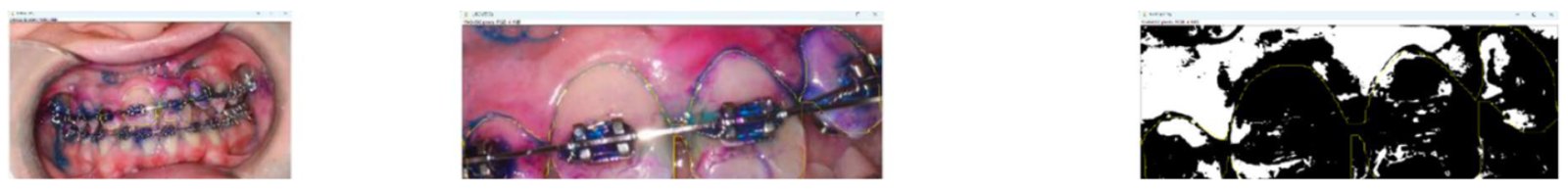			797,571	273,645	25.5
**2**	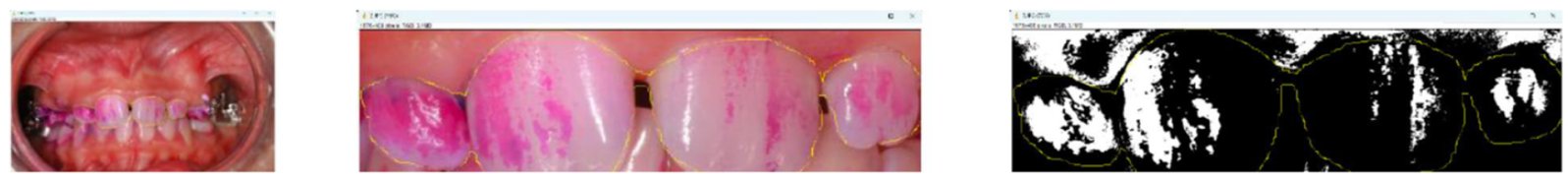			655,898	161,990	19.8
**3**	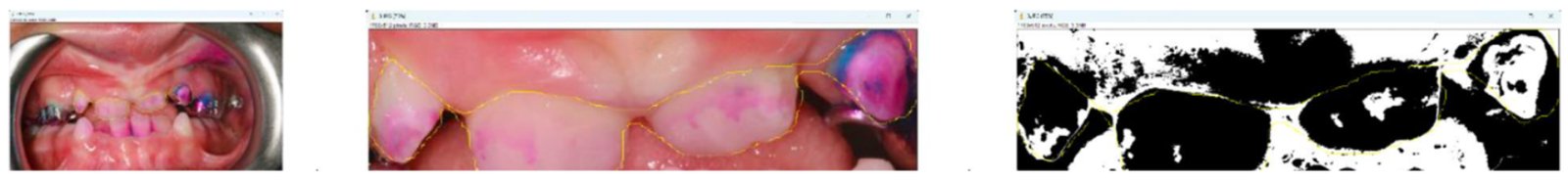			539,565	330,835	38
**4**	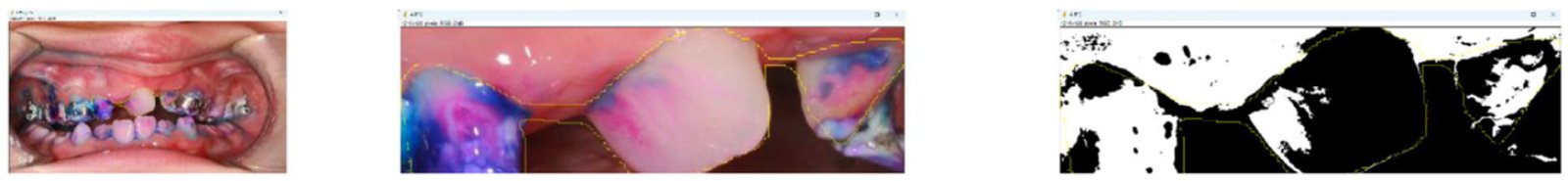			325,175	195,273	50
**5**	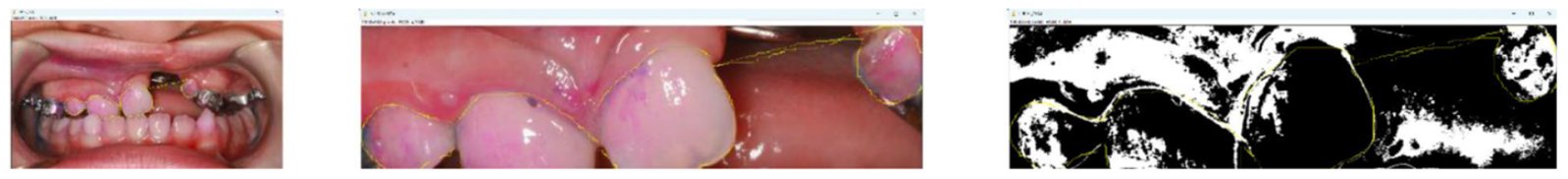			798,713	323,191	28.8
**6**	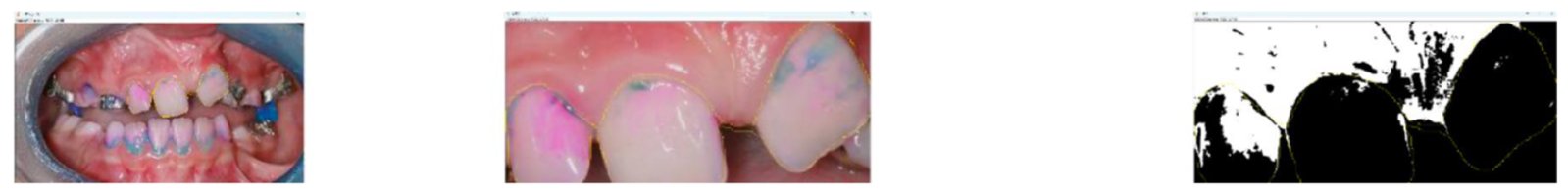			662,238	306,306	42.1
**7**	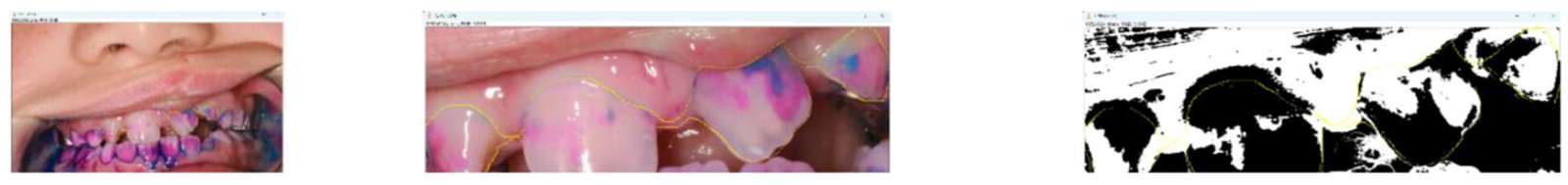			551,748	479,100	46.5
**8**	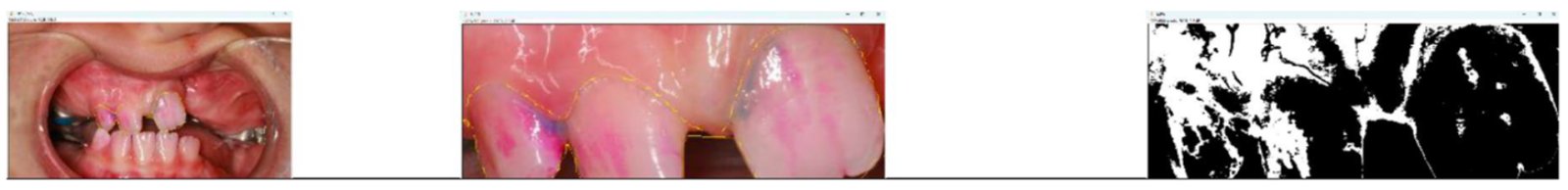			595,805	145,075	26.1
**9**	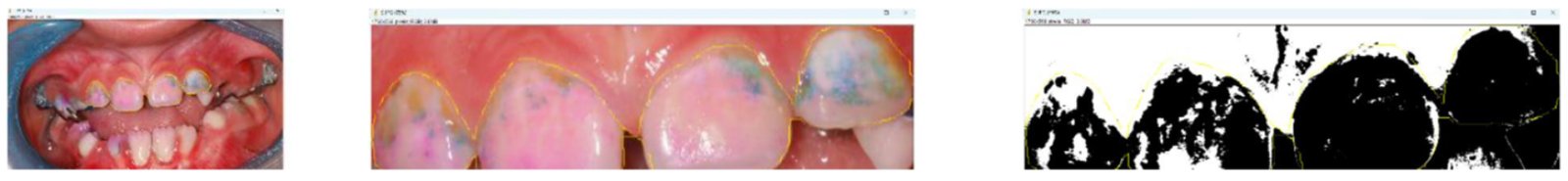			629,108	370,572	37.1
**12**	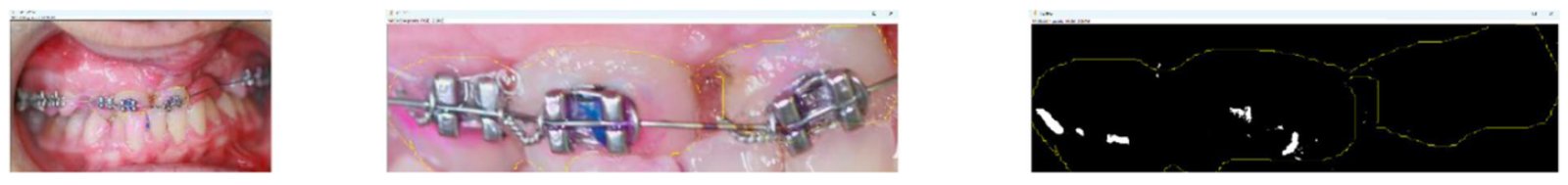			729,182	6658	1.2
**14**	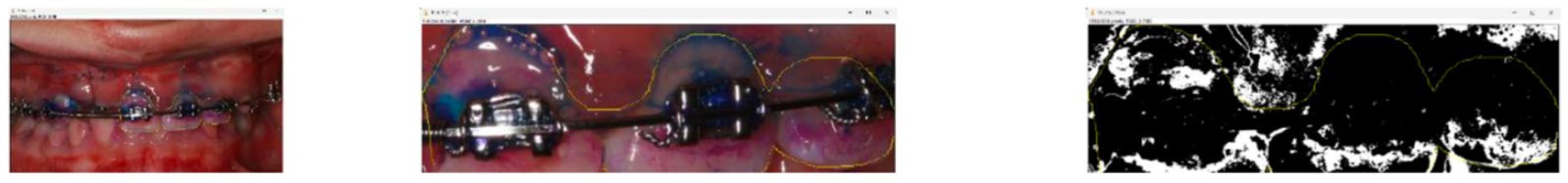			796,271	171,665	23.6
**16**	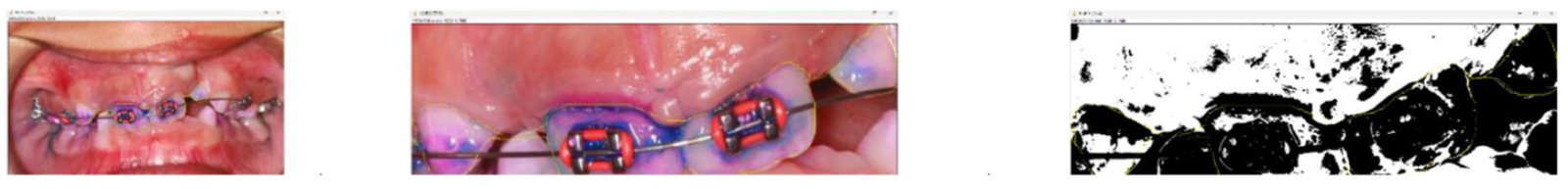			625,456	717,776	53.4
**19**	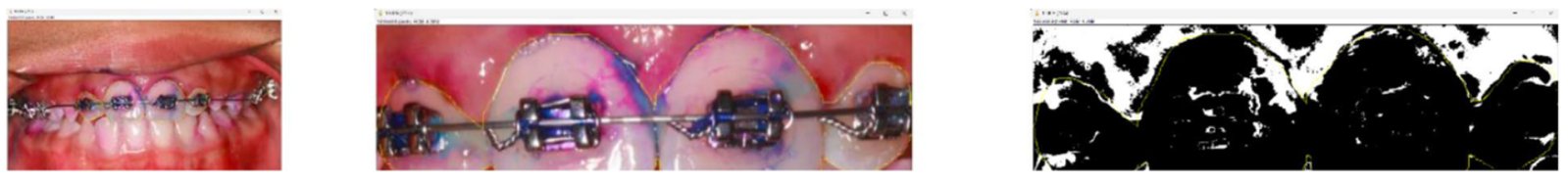			880,668	235,620	21.1

## Data Availability

The datasets generated during and/or analyzed during the current study are available from the corresponding author on request.
